# Autophagy-related protein MoAtg14 is involved in differentiation, development and pathogenicity in the rice blast fungus *Magnaporthe oryzae*

**DOI:** 10.1038/srep40018

**Published:** 2017-01-09

**Authors:** Xiao-Hong Liu, Ya-Hui Zhao, Xue-Ming Zhu, Xiao-Qing Zeng, Lu-Yao Huang, Bo Dong, Zhen-Zhu Su, Yao Wang, Jian-Ping Lu, Fu-Cheng Lin

**Affiliations:** 1 State Key Laboratory for Rice Biology, Biotechnology Institute, Zhejiang University, Hangzhou, 310058, China; 2 College of Life Sciences, Zhejiang University, Hangzhou, 310058, China; 3 Agricultural Technology Extension Center, Zhejiang University, Hangzhou, 310058, China; 4 State Intellectual Property Office of the People’s Republic of China, Beijing, 100080, China; 5 State Key Laboratory of Breeding Base for Zhejiang Sustainable Pest and Disease Control, Institute of Virology and Biotechnology, Zhejiang Academy of Agricultural Sciences, Hangzhou 310021, Zhejiang Province, China

## Abstract

Autophagy is the major intracellular degradation system by which cytoplasmic materials are delivered to and degraded in the vacuole/lysosome in eukaryotic cells. MoAtg14 in *M. oryzae*, a hitherto uncharacterized protein, is the highly divergent homolog of the yeast Atg14 and the mammal BARKOR. The *MoATG14* deletion mutant exhibited collapse in the center of the colonies, poor conidiation and a complete loss of virulence. Significantly, the Δ*Moatg14* mutant showed delayed breakdown of glycogen, less lipid bodies, reduced turgor pressure in the appressorium and impaired conidial autophagic cell death. The autophagic process was blocked in the Δ*Moatg14* mutant, and the autophagic degradation of the marker protein GFP-MoAtg8 was interrupted. GFP-MoAtg14 co-localized with mCherry-MoAtg8 in the aerial hypha. In addition, a conserved coiled-coil domain was predicted in the N-terminal region of the MoAtg14 protein, a domain which could mediate the interaction between MoAtg14 and MoAtg6. The coiled-coil domain of the MoAtg14 protein is essential for its function in autophagy and pathogenicity.

*Magnaporthe oryzae*, the causal agent of rice blast, has been chosen as a model to study the interaction between fungi and plants. Common to many other plant pathogenic fungi, *M. oryzae* elaborates a signature penetration structure, the appressorium, to infect its host^1^,^2^,^3^. The whole infectious cycle of *M. oryzae*, from surface recognition, adherence, and appressorium formation to infectious growth and pathogenicity, is closely related to signal transduction pathways and protein degradation processes. The typical signal transduction pathways, including mitogen activated protein kinase (MAPK), cyclic adenosine monophosphate (cAMP), and calcium signal transduction pathways^4^,^5^,^6^,^7^, and the protein degradation processes, including autophagy^8^,^9^,^10^, ubiquitin mediated protein degradation^11^,^12^,^13^ and calpains^14^,^15^, have been confirmed to play significant roles in cell cycling, cellular differentiation and pathogenesis of *M. oryzae*.

Autophagy is an intracellular degradation system that delivers cytoplasmic materials to the lysosome/vacuole during development and in response to nutrient stress in eukaryotic cells^16^. The autophagy process was verified as an essential catabolic process that plays important roles in cell stress management and nutrient homeostasis. The differentiation, cell vitality, and infectious structures are impaired when the autophagy process is blocked. Current studies have shown that the Atg proteins required for autophagy constitute the following five functional groups: (i) the Atg1 kinase complex (Atg1-13-17-29-31), (ii) the Atg9 membrane protein recycling system, (iii) the class III phosphatidylinositol 3-kinase (PI3-K) complex (Atg6-Atg14-Vps15-Vps34) (hereafter, PI3-K indicates the class III PI3-K), (iv) the Atg12-Atg5-Atg16 protein conjugation system, and (v) the Atg8 lipid conjugation system^16^,^17^. For the past two decades, the core proteins of every autophagy group have been verified to function in *M. oryzae*, for example, MoAtg1 in the Atg1 kinase complex^18^, MoAtg9 in the Atg9 membrane protein recycling system^19^, MoAtg5 in the conjugation system^20^, and MoAtg4 and MoAtg8 in the lipid conjugation system^8^,^21^,^22^. Extensive research has been performed on other plant pathogenic fungi, including *Fusarium*^23^,^24^,^25^, *Colletotrichum*^26^,^27^,^28^, *Ustilago*^29^,^30^, and *Verticillium*^31^. However, there are few studies of the PI3-K complex in plant pathogenic fungi.

PI3-K is essential for both autophagy and vacuolar protein sorting in yeast and mammals^32^,^33^,^34^. Atg14 is the autophagy-specific subunit of the PI3-K complex and a key factor in determining the function of the PI3-K complex. Atg14 is present on the preautophagosomal structure (PAS) and is thought to function at an early stage of autophagosome formation. Initially, Atg14 was only described in yeast species^35^. Recently, a protein with an extremely low similarity to yeast Atg14 was identified in humans and named ATG14/ATG14L/BARKOR^36^,^37^. In *M. oryzae*, a conventional BLAST database search failed to identify a homolog of Atg14. Using a combination of Pfam domain analysis, position specific iterated (PSI)-BLAST and the pattern hit-initiated basic local alignment search tool (PHI-BLAST), MoAtg14 was identified in the database of *M. oryzae*. The conserved coiled-coil protein MGG_03698, designated MoAtg14 in the genome of *M. oryzae*, was shown to have very weak similarity to ScAtg14 and HsAtg14.

To date, no experimental evidence has emerged to explain the functions of the homolog of Atg14 in *M. oryzae*. In our research, we found that MoAtg14 was conserved in the filamentous ascomycetes. Deletion of MoAtg14 resulted in defects in conidiation, breakdown of glycogen and lipid bodies, turgor pressure of appressoria, pathogenicity, and the autophagy process. Subcellular localization and microscopic examination indicated that MoAtg14 is present on the PAS and plays key roles at the stage of autophagosome formation. The localization of MoAtg8 was impaired in the MoAtg14 deletion mutant. The conserved coiled-coil domain of MoAtg14 plays critical roles in *M. oryzae*.

## Results

### Identification of MoAtg14 in *M. oryzae*

Pfam domain analysis of the *M. oryzae* proteome was used to identify the proteins. The integrated module of the Pfam domain was searched with the CLC Genomics Workbench (Qiagen, Germany) using the default parameters. The Pfam database used in the analysis was version 27. MGG_03698 and MGG_13375 were found to contain the conserved domain PF10186. We reanalyzed protein databases at the NCBI by position specific iterated (PSI-BLAST) and pattern hit-initiated basic local alignment search tool (PHI-BLAST) using both yeast and human Atg14. The conserved coiled-coil protein MGG_03698 in the genome of *M. oryzae* was confirmed to have weak similarity to ScAtg14 and HsAtg14 and was designated MoAtg14. The other protein, MGG_13375, showed more similarity to mammalian UVRAG proteins (a counterpart of the mammalian Vps38)^37^,^38^, implying that MGG_13375 might represent the fungal ortholog of Vps38.

**Figure 1:**
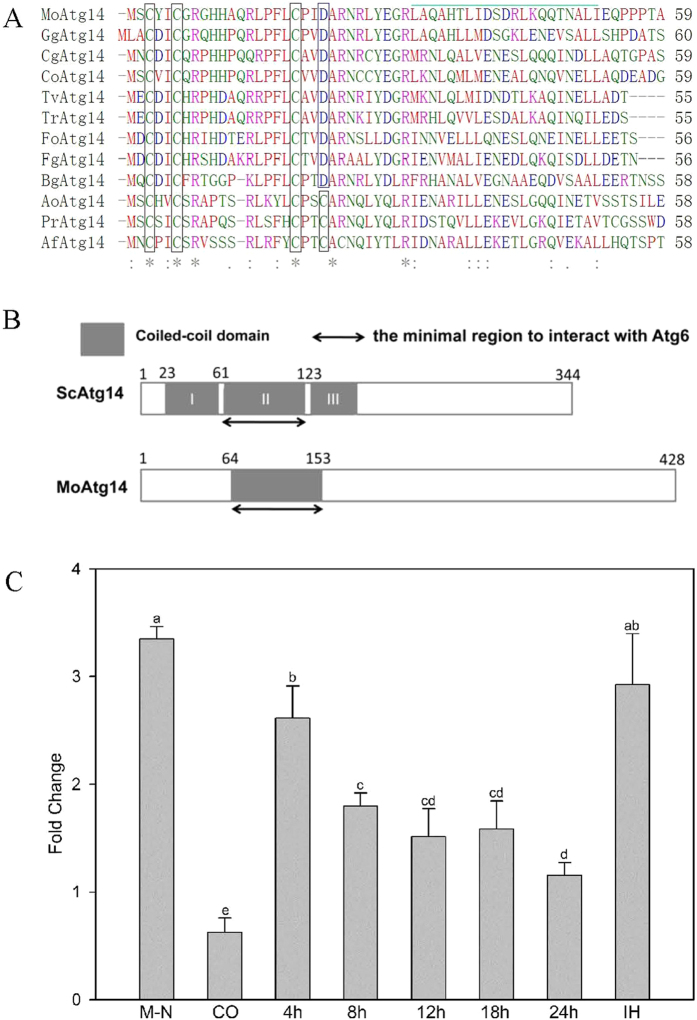
(**A**) The amino acid sequence of the N-terminal motif containing the conserved cysteine residues in the ascomycetes fungi. The conserved cysteine residues are in the box. The green line indicates the start of the conserved coiled-coil region. GgAtg14, accession No. XP_009224438; CgAtg14, accession No. EQB48915; CoAtg14, accession No. ENH80301; TvAtg14, accession No. XP_013959553; TrAtg14, accession No. XP_006966865; FoAtg14, accession No. EMT61395; FgAtg14, accession No. XP_011316371; BgAtg14, accession No. EPQ63265; AoAtg14, accession No. BAE65502; AfAtg14, accession No. XP_747209; PrAtg14, accession No. CDM36188. (**B**) The domains of the yeast ScAtg14 and *M. oryzae* MoAtg14. Boxes in grey indicate the coiled-coil domains. (**C**) The expression profiles of the *MoATG14* gene in development, pathogenicity and starvation stress. qRT-PCR assays were carried out with RNA samples obtained from different stages of the wild-type strain Guy11, including vegetative hyphae, conidia (CO), appressoria, invasive hyphae (IH) and nitrogen starved hyphae (MM-N). Gene expression levels were normalized using the β-tubulin gene as an internal standard. Data are representative of at least two independent experiments with similar results, and the error bars represent the standard deviations of three replicates (P < 0.01). Different letters indicate a significant difference.

Analysis of the domain of MoAtg14 showed that it contains a conserved Cys-rich motif at its N-terminus (Fig. 1A). The motif is also present in yeast and human Atg14, and it displays high levels of similarity to homologs in other filamentous ascomycetes, including *Gaeumannomyces graminis* (55% identity), *Colletotrichum orbiculare* (50% identity), *Fusarium graminearum* (46% identity) and *Blumeria graminis* (39% identity). To verify the high similarity of MoAtg14 with Atg14 in other ascomycetes, we selected *F. graminearum* Atg14 (FgAtg14) and *Trichoderma reesei* Atg14 (TrAtg14) to complement the Δ*Moatg14* mutant. Reintroduction of FgAtg14 or TrAtg14 to the mutant, the defects of the Δ*Moatg14* mutant could be recovered completely (Figure S1).

It has been reported that three predicted coiled-coil domains exist in the N-terminal half of yeast Atg14. These coiled-coil domains are sufficient to support the autophagic ability as revealed by deletion analysis of yeast Atg14. The second coiled-coil domain of yeast Atg14 interacted with Atg6^35^,^39^. However, only one coiled-coil domain exists in the N-terminus of MoAtg14 in *M. oryzae* as predicted by COILS (http://www.ch.embnet.org/software/COILS_form.html) (Figs 1B and S2). Our research revealed the detailed functions of MoAtg14, as described below. In addition, MoVps38 contains a coiled-coil domain (Figure S3). Unfortunately, we were not able to isolate a null mutant of MoVps38.

To determine the expression profiles of the *MoATG14* gene during development (in vegetative hyphae, conidia, and appressoria), pathogenicity (in infective hyphae) and starvation stress (in nitrogen starved hyphae), expression was evaluated using qRT-PCR assays (Fig. 1C). Compared with the expression level of *MoATG14* in vegetative hyphae, in the nitrogen starved hyphae, the expression level was more than 3-fold higher. In addition, the expression level of *MoATG14* was more than 2-fold higher in 4 h -appressoria and invasive hyphae than in vegetative hyphae.

### Deletion of *MoATG14* in *M. oryzae*

**Figure 2:**
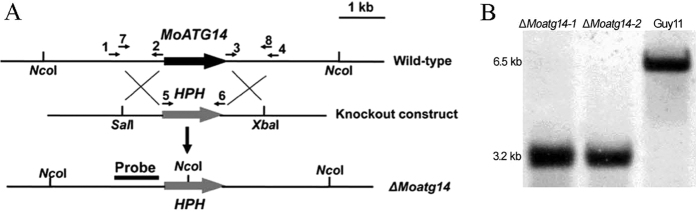
Targeted gene deletion of *MoATG14* in *M. oryzae*. (**A**) The *MoATG14* locus and gene deletion vector. Arrows 1–8 indicate the primers ATG14up-1/2, ATG14dn-1/2, HPH-1/2 and ATG14-N1/2. (**B**) Southern blot analysis of Δ*Moatg14* mutants 1 and 2, and the wild-type strain Guy11. Genomic DNA was digested with *Nco*I and separated on a 0.7% agarose gel. The DNA was hybridized with the probe (indicated in **A**) amplified from genomic DNA of Guy11.

To determine the biological functions of *MoATG14* in *M. oryzae*, we constructed a deletion mutant by targeted gene replacement using ATMT (Fig. 2A). Southern blot assays were performed to confirm single-copy genomic integration and exclude additional ectopic integrations. An approximately 3.2 kb band was detected in Δ*Moatg14* mutants, in contrast to an approximately 6.5 kb band in the wild-type strain Guy11 (Fig. 2B). Two Δ*Moatg14* mutants showed comparable phenotypes, and Δ*Moatg14-1* was chosen for further studies. Complementation assays of Δ*Moatg14-1* were carried out, and the transformant Moatg14c, which contained a full-length gene copy of *MoATG14*, was selected for further studies.

### MoAtg14 is required for hyphal development, conidiogenesis and pathogenicity

**Figure 3:**
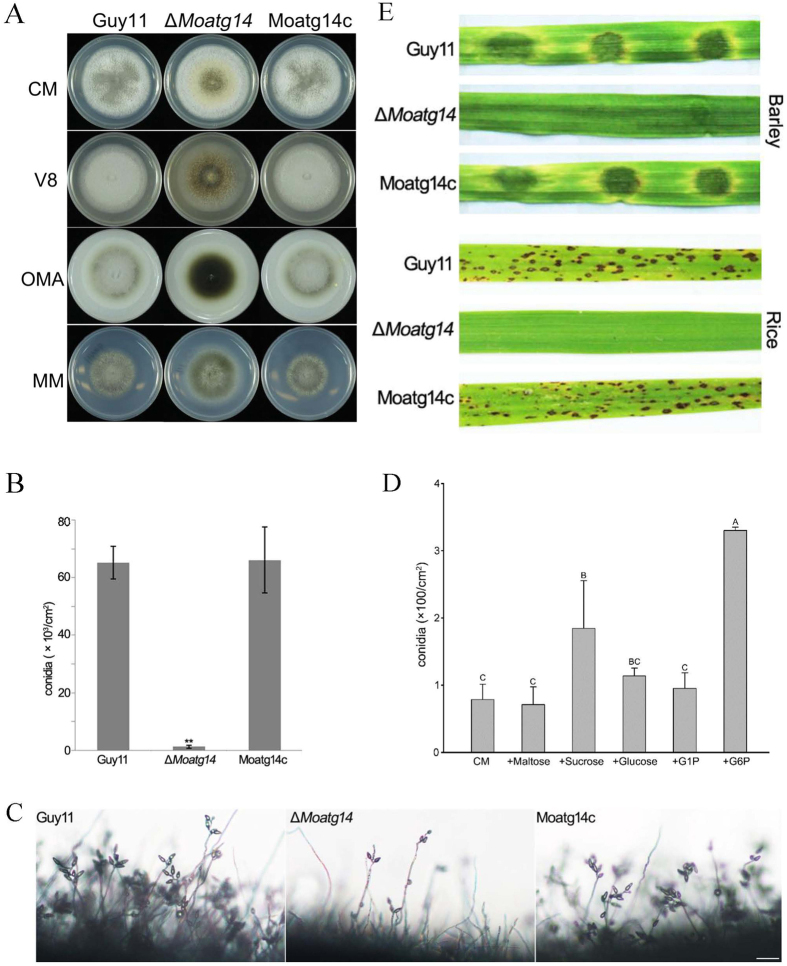
Characteristics of *M. oryzae* strains. (**A**) Guy11, the Δ*Moatg14* mutant, and the complemented strain Moatg14c were grown on CM, V8, OMA, and MM medium for 8 days. (**B**) Few conidia were produced by the Δ*Moatg14* mutant, in contrast to Guy11 and Moatg14c. Error bars represent one standard deviation (P < 0.01). Different letters indicate a significant difference in the conidiation of the Δ*Moatg14* mutant, Guy11, and Moatg14c. (**C**) Development of conidia on conidiophores observed under cover slips with a light microscope 24 h after induction of conidiation. Few conidia developed in the Δ*Moatg14* mutant. Scale bar = 50 μm. (**D**) Conidiation in the Δ*Moatg14* mutant grown on CM medium and CM medium supplemented with 10 g/L maltose, 6.25 g/L sucrose, 10 g/L glucose, 1 mM G1P and 0.5 mM G6P. Error bars represent one standard deviation (P < 0.01). Different letters indicate a significant difference. (**E**) The *MoAtg14* deletion mutant is nonpathogenic. Disease symptoms on cut leaves of barley inoculated with mycelial plugs from Guy11, the Δ*Moatg14* mutant, and Moatg14c. Typical leaves were photographed 4 days after inoculation. Two-week-old rice seedlings were inoculated by spraying with 1 × 10^5^ conidia/ml conidia suspensions from Guy11, the Δ*Moatg14* mutant, and Moatg14c. Lesion formation on the rice leaves was evaluated 7 days after inoculation.

On CM plates, the Δ*Moatg14* mutant showed vegetative growth similar to that of the wild-type strain Guy11, and the complemented strain Moatg14c. However, the Δ*Moatg14* mutant showed sparse hyphae with necrotic centrality, especially on the V8 and OMA media, in contrast to the dense hyphae of Guy11 and Moatg14c (Fig. 3A). On a 10-day-old CM plate, the number of conidia produced by the Δ*Moatg14* mutant was only 1/50 of the number produced by Guy11 (Fig. 3B). Microscopic examination revealed that small numbers of conidia were observed on the conidiophores of the Δ*Moatg14* mutant at 24 h post-conidial induction after an 8-day incubation (Fig. 3C). These observations suggested that the *MoATG14* gene plays key roles in hyphal development and conidiogenesis.

It has been reported that alternate carbon sources (mostly sugars) can suppress the conidiation defects of the Δ*Moatg8* mutant^22^. To explore the effects of alternate carbon sources on the conidiation capability of the Δ*Moatg14* mutant, the conidia of the mutant were collected and counted after 10 days on complete medium (CM) supplemented with additional maltose, sucrose, glucose, glucose 1-phosphate (G1P), or glucose 6-phosphate (G6P). As expected, the addition of G6P significantly restored conidiation in the Δ*Moatg14* mutant (Fig. 3D). There were 3 times more conidia on CM supplemented with G6P than on CM. In addition, the number of conidia on CM supplemented with sucrose was approximately 2-fold that on CM. These data confirmed that alternate sugar sources could relieve the conidiation defects resulting from the loss of autophagy.

In infection assays with two susceptible hosts (rice and barley), the Δ*Moatg14* mutant failed to penetrate either host. Disease symptoms were not observed when mycelial plugs of Δ*Moatg14* were inoculated onto barley. In contrast, the wild-type strain Guy11 and the complemented strain Moatg14c, induced susceptible lesions. Similarly, no spindle-shaped lesions were observed on rice inoculated with a Δ*Moatg14* conidial suspension (1 × 10^5^ conidia/ml). These data indicated that the *MoATG14* gene is important for plant infection (Fig. 3E).

### MoAtg14 is required for glycogen mobilization, quantity of lipid bodies, and the turgor pressure of appressoria

**Figure 4:**
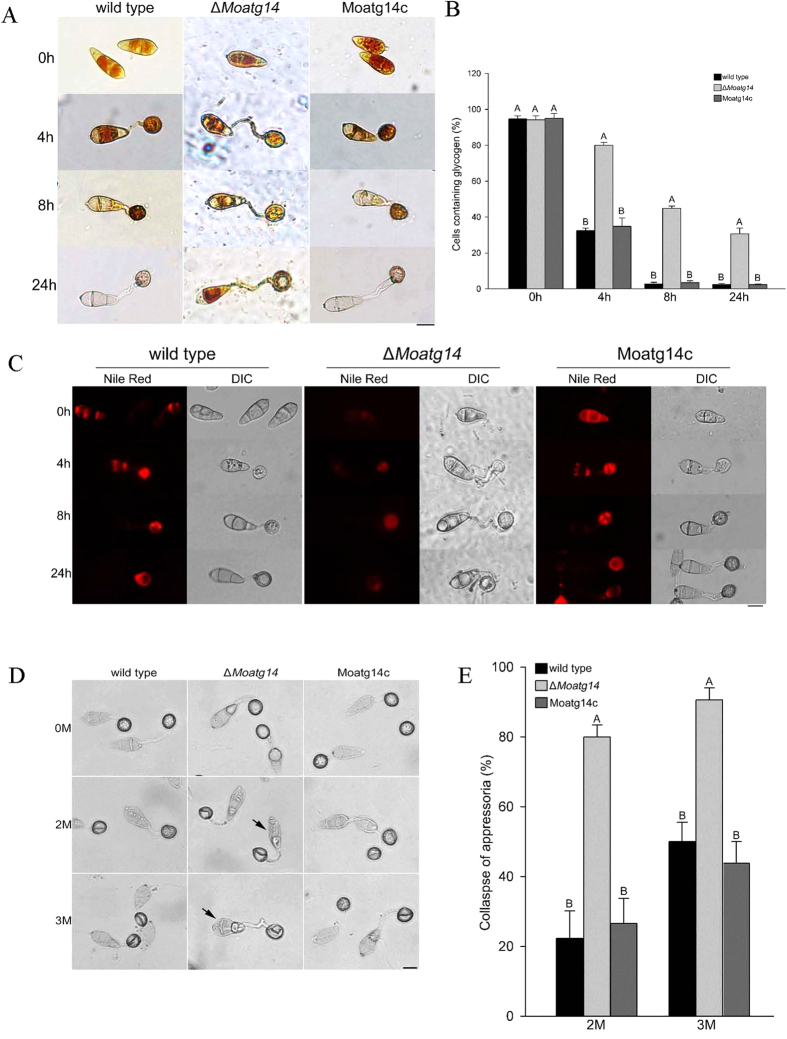
Conidia of the wild-type strain, the Δ*Moatg14* mutant, and the complemented mutant Moatg14c were allowed to form appressoria on plastic coverslips at 0 h, 4 h, 8 h and 24 h after inoculation. (**A**) Cellular distribution of glycogen. Samples were stained with KI/I_2_ solution. Microscopically, the glycogen appears as dark brown deposits. Scale bar = 10 μm. (**B**) The proportion of the conidial cells containing glycogen stained by KI/I_2_ solution during appressorium development in the Guy11, the Δ*MoAtg14* mutant, and Moatg14c. Error bars represent one standard deviation (P < 0.01). Different letters indicate a significant difference. (**C**) Cellular distribution of lipid droplets. Samples were stained with Nile red and observed in the dark with UV epifluorescence. The lipid droplets show a red signal fluorescence. Scale bar = 10 μm. (**D**) Collapse of appressoria. Conidia were allowed to form appressoria on plastic coverslips 24 h after inoculation, and the collapsed appressoria were assessed after exposure to 2 M or 3 M glycerol solution for ten minutes. Arrows indicate the collapsed portions of the conidia. Scale bar = 10 μm. (**E**) The turgor pressure of the appressoria was measured by incipient cytorrhysis assays. The proportion of the collapsed appressoria after exposure to 2 M or 3 M glycerol solution for ten minutes are shown. Error bars represent one standard deviation (P < 0.01). Different letters indicate a significant difference.

Glycogen and lipids are the most abundant storage products in *M. oryzae* conidia. Glycogen is rapidly degraded during conidial germination, and lipid bodies are transported to the developing appressoria and degraded at the onset of turgor generation^40^. Previous studies showed that the breakdown of glycogen and lipid bodies was delayed in the autophagy-deficient mutants (Δ*Moatg1*, Δ*Moatg4*, Δ*Moatg5*, Δ*Moatg8*, and Δ*Moatg9*)^8^,^18^,^19^,^20^,^21^,^22^. Therefore, the cellular distribution of glycogen and lipid bodies was examined during appressorium development in the Δ*Moatg14* mutant. The wild-type strain and the Δ*Moatg14* mutant show similar distributions of glycogen in conidia (Fig. 4A, 0 h). With the development of conidia, the glycogen in the conidia was degraded gradually in the wild-type strain. After 24 h, a very small proportion of the wild-type conidia contained glycogen. In contrast, a high proportion of the Δ*Moatg14* conidia contained glycogen from the stage of the germ tube to mature appressoria (Fig. 4A). At 24 h, 30% of the cells of the Δ*Moatg14* conidia contained glycogen compared to 2% of the cells of the wild-type conidia (Fig. 4B). These data indicated that the breakdown of glycogen was significantly retarded in the Δ*Moatg14* mutant. Next, we investigated the distribution of lipid bodies by Nile red staining in the Δ*Moatg14* mutant from conidia to appressoria (Fig. 4C). The Δ*Moatg14* mutant showed faint fluorescence in contrast to the bright fluorescence of the wild type.

Glycogen mobilization was delayed significantly and lipid bodies were reduced in the Δ*Moatg14* mutant. We examine the turgor pressure of the Δ*Moatg14* appressoria using the incipient cytorrhysis assays. As shown in Fig. 4D, the appressoria of Δ*Moatg14* severely collapsed in 2 M glycerol. In the Δ*Moatg14* mutant, 80% of the appressoria collapsed at 24 h compared to 22% and 26% of the appressoria of the wild-type and complemented strains, respectively. In 3 M glycerol, a high proportion of the Δ*Moatg14* appressoria remained severely collapsed (Fig. 4D,E). In summary, the turgor pressure of the Δ*Moatg14* appressoria was significantly lower than in the wild-type or the complemented strain.

### MoAtg14 is required for autophagy and conidial autophagic cell death

**Figure 5:**
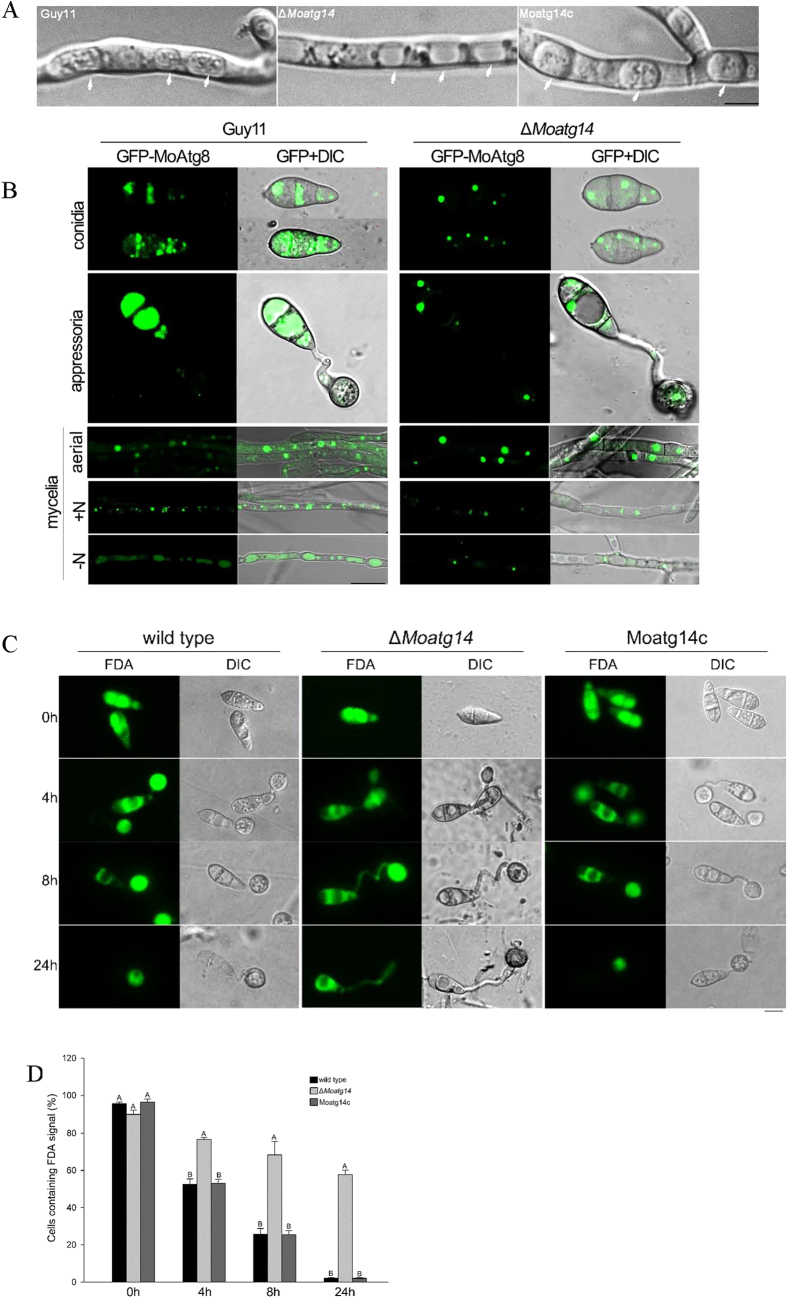
Autophagy was blocked in the Δ*Moatg14* mutant. (**A**) Autophagy was triggered in the starved mycelia. Numerous autophagic bodies were detected in the vacuoles of Guy11 and Moatg14c under starvation conditions. The mycelia were treated with 4 mM PMSF under nitrogen starvation for 4 h. No autophagic bodies were evident in Δ*Moatg14* mutant vacuoles under the same conditions. Arrows indicate the vacuoles. (**B**) Normal GFP-MoAtg8 localization was impaired in the Δ*Moatg14* mutant. Conidia were collected from the Guy11 and Δ*MoAtg14* strains expressing GFP-MoAtg8. Appressoria: 1 × 10^4^ conidia of Guy11 and Δ*MoAtg14* expressing GFP-MoAtg8 were inoculated on the hydrophobic cover slip and incubated for 8 h. Mycelia: aerial hyphae of Guy11 and Δ*MoAtg14* expressing GFP-MoAtg8. The strains Guy11 and Δ*MoAtg14* expressing GFP-MoAtg8 were grown in liquid CM medium at 25 °C for 48 h (N+), and shifted to liquid MM-N medium with 4 mM PMSF for 4 h (N−). Scale bar = 10 μm. (**C**) Conidial autophagic cell death assays of strains. The conidial cells of the wild-type strain and Moatg14c showed conidial autophagic cell death during appressoria development. The Δ*MoAtg14* mutant had defects in conidial autophagic cell death. (**D**) The proportion of the conidial cells containing an FDA signal during the development of the Guy11, Δ*MoAtg14* and Moatg14c strains. Error bars represent one standard deviation (P < 0.01). Different letters indicate a significant difference.

In yeasts, Atg14 is a key player in orchestrating autophagy^35^. To determine whether the loss of *MoATG14* affects autophagy in *M. oryzae*, vacuoles of hyphal cells were examined with starvation induction assays. When cultured in sterile distilled water in the presence of 2 mM PMSF for 4 h, no autophagic bodies were detected in the lumen of vacuoles in the Δ*Moatg14* mutant hyphae, whereas a large number of autophagic bodies existed in the vacuole lumen of Guy11 and Moatg14c cultures (Fig. 5A).

To identify other functions of *MoATG14* in autophagy, GFP-MoAtg8 trafficking assays were performed. Under normal conditions in the wild-type, Guy11, GFP-MoAtg8 was localized to punctate structures that were proximal to the vacuoles, whereas in nitrogen starvation conditions, GFP fluorescence showed an even distribution in the lumen of the vacuoles (Fig. 5B, Guy11). In contrast, in the Δ*Moatg14* mutant, GFP-MoAtg8 failed to localize in the vacuoles upon nitrogen starvation (Fig. 5B, Δ*Moatg14*). Accumulation of GFP in vacuoles was absent in every developmental phase of Δ*MoAtg14*, including conidia, appressoria and mycelia (Fig. 5B, Δ*Moatg14*). Multiple punctate structures of GFP-MoAtg8 were assembled into larger dots proximal to the vacuoles, and no green signals were observed in the vacuole lumen after 4 h of nitrogen starvation in the Δ*Moatg14* mutant. Conversely, GFP accumulated in the vacuoles of the wild-type, Guy11, from conidia to mature appressoria (Fig. 5B, Guy11). The results indicated that GFP-MoAtg8 trafficking was impaired in the Δ*Moatg14* mutant.

Because Δ*Moatg14* failed to deliver GFP-Atg8 to the vacuoles upon nitrogen starvation, GFP-MoAtg8 proteolysis assays were performed to monitor the autophagy process. Under normal conditions, the 41-kDa GFP-MoAtg8 fusion band was detected in anti-GFP western blotting in the wild-type, Guy11. When cultured hyphae were shifted to starvation conditions, the levels of free GFP increased with time, apparently at the expense of full-length GFP-MoAtg8. In contrast, only full-length GFP-MoAtg8 was detected in the Δ*Moatg14* mutant (Figure S4). The proteolysis of GFP-MoAtg8 was completely prevented in the Δ*Moatg14* mutant.

All these data confirmed that the autophagy pathway was blocked in the Δ*Moatg14* mutant. Next, we examined the conidial cell death in the Δ*Moatg14* mutant. The cells were strained by fluorescein diacetate (FDA), a viability stain for cells. The green fluorescence proved that the cells were still alive. As shown in Fig. 5D, a high proportion of the cells of the Δ*Moatg14* conidia showed green fluorescence after 24 hpi on the plastic coverslip (Fig. 5C,D). In contrast, very few cells of the wild-type conidia showed fluorescence after 24 hpi on the plastic coverslip. These results indicated that the Δ*Moatg14* mutant did not show conidial cell death.

### MoAtg14 is present in PAS

**Figure 6:**
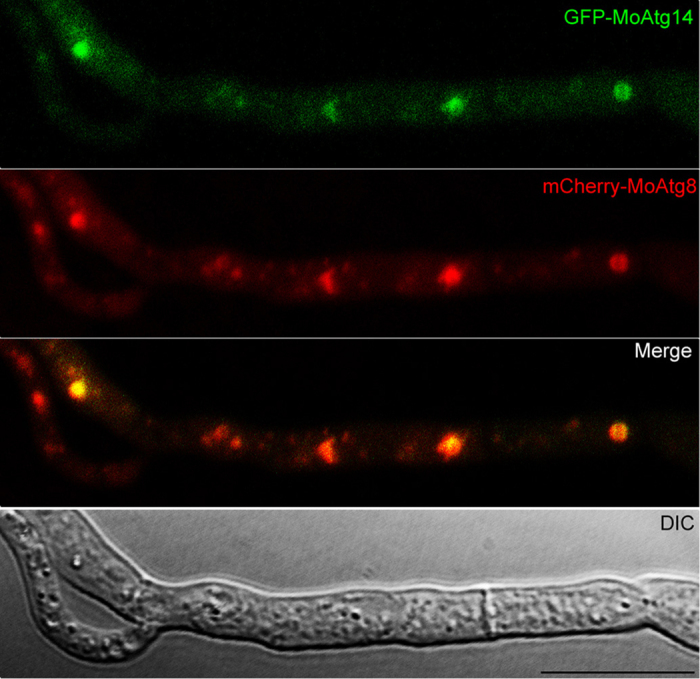
Co-localization of GFP-MoAtg14 and mCherry-MoAtg8. Scale bar = 10 μm.

To determine the location of MoAtg14, we transformed the GFP-MoAtg14 fusion expression vector into the Δ*Moatg14* mutant. In the transformants, the defects of Δ*Moatg14* were complemented, which indicated that the GFP-MoATG14 fusion protein is functional (data not shown). Faint fluorescence could be detected in the cytoplasm of conidia, mycelia and appressoria (Figure S5). However, in aerial hyphae, which are without external nutrients, GFP-MoAtg14 appeared as punctuate dots. Our previous study showed that mCherry-MoAtg8 localized at multiple PAS sites in *M. oryzae*^19^. To investigate the localization of the GFP-MoAtg14 dots, mCherry-MoAtg8 was transformed into the mutant containing GFP-MoAtg14. We found that puncta of GFP-MoAtg14 co-localized with mCherry-MoAtg8, leaving other green signals distributed throughout the entire cytoplasm (Fig. 6). The co-localization of GFP-MoAtg14 and mCherry-MoAtg8 suggested that MoAtg14 is present in the PAS in *M. oryzae*.

### The coiled-coil domain of MoAtg14 plays crucial roles in *M. oryzae*

**Figure 7:**
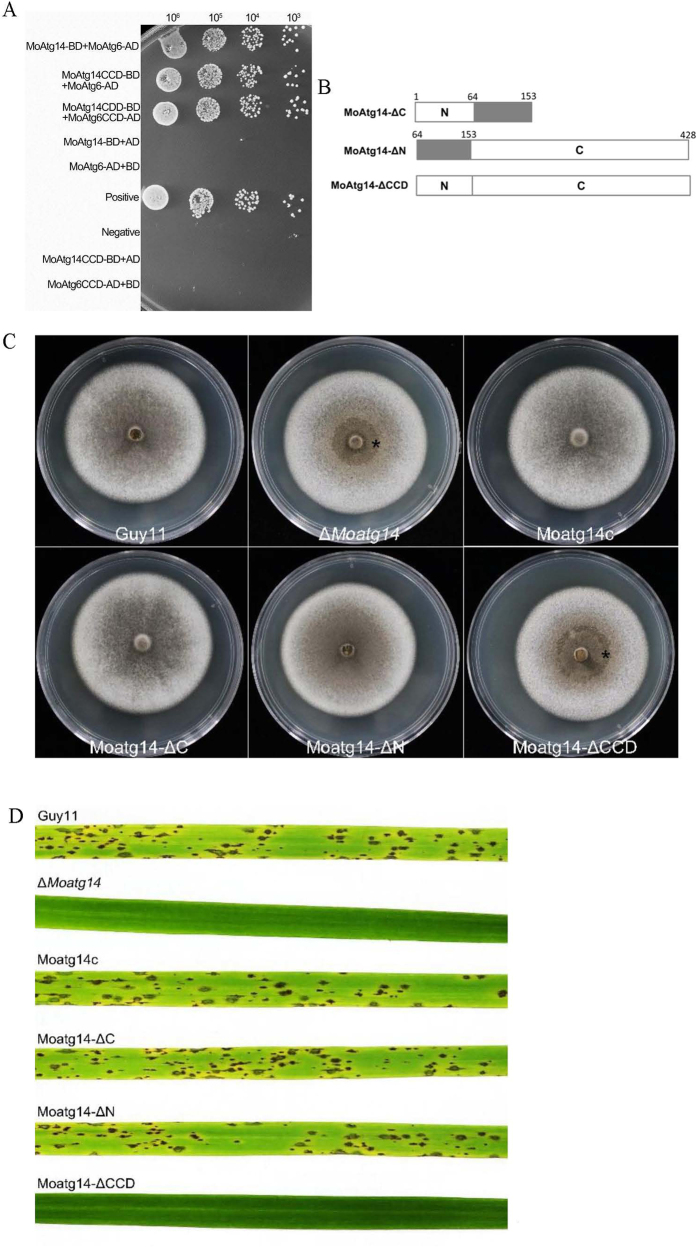
(**A**) Yeast two-hybrid assays. The interactions between MoAtg14 and MoAtg14 CCD as bait and MoAtg6 and MoAtg6 CCD as the prey were assessed. Yeast transformants grown on the SD/-Ade/-Leu/-Trp/-His plates were assayed for β-galactosidase activity. (**B**) Schematic representation of deleted variants of MoAtg14. (**C**) The coiled-coil domain in MoAtg14 is essential to maintain normal colony morphology. Strains were grown on CM for 10 days. The asterisk indicates the collapse of the colony. (**D**) The coiled-coil domain in MoAtg14 is essential to maintain normal pathogenicity. Disease symptoms of rice inoculated with 1 × 10^5^ conidia from Guy11, the Δ*Moatg14* mutant, Moatg14c, Moatg14-ΔC, Moatg14-ΔN, and Moatg14-ΔCCD. The lesions formed on the rice were photographed 7 days after inoculation.

As previously described, Atg14/Barkor physically interacts with Atg6/Beclin1 in yeasts and mammals^37^,^38^. To determine whether MoAtg14 interacts with MoAtg6 in *M. oryzae*, yeast two-hybrid assays were performed. The results showed that yeast cells containing the full-length MoAtg14 (prey) and MoAtg6 (bait) on two-hybrid plasmids could grow well on the Ade-His-Leu-Trp-minus-selective plates (Fig. 7A). The coiled-coil domain of MoAtg14 (MoAtg14^64–153^) was cloned into the pGADT7 vector to generate the prey vector pAD-MoAtg14^64–153^. Cells containing pAD-MoAtg14^64–153^ could grow on the Ade-His-Leu-Trp-minus selective plates in combination with the full-length MoAtg6 (BD) vector. The N-terminus (MoAtg14^1–73^) and C-terminus (MoAtg14^144–428^) were cloned into the pGADT7 vector and co-transformed into AH109 cells. However, the cells could not grow on the Ade-His-Leu-Trp-minus selective plates in combination with the full-length MoAtg6 (bait) vector (Fig. 7A). We obtained similar results when we used the coiled-coil domain of MoAtg6 as bait. The coiled-coil domain of MoAtg6 interacted with the full-length MoAtg14 or MoAtg14^64–153^. Cells could grow on the Ade-His-Leu-Trp-minus-selective plates. This result indicated that the coiled-coil domain of MoAtg14 was required for the interaction with MoAtg6. The primary function of MoAtg14 is mediated by protein-protein interactions of the coiled-coil domain.

To investigate the function of the coiled-coil domain of MoAtg14 in *M. oryzae*, pMoAtg14-ΔN, pMoAtg14-ΔC and pMoAtg14-ΔCCD vectors were constructed to transform into the Δ*Moatg14* mutant separately (Fig. 7B). The results showed that the morphology of the colonies, conidiation, pathogenicity and autophagy recovered when we introduced pMoAtg14-ΔN or pMoAtg14-ΔC. However, pMoAtg14-ΔCCD could not recover the defects of the Δ*Moatg14* mutant (Fig. 7C,D). The phenotypes of the MoAtg14-ΔCCD mutant were similar to that of the Δ*Moatg14* mutant (Fig. 7C,D), which indicated that the coiled-coil domain is the core of MoAtg14.

## Discussion

Autophagy has been extensively studied in *M. oryzae*. In this research, we focused on MoAtg14, an important subunit of the PI3-K complex, which is essential for autophagy. Analysis using a gene knockout indicated that MoAtg14 is essential for autophagy and plays key roles in conidiation, the breakdown of glycogen, numbers of lipid bodies, pathogenicity, conidial autophagic cell death, and autophagosome formation. The predicted coiled-coil domain of MoAtg14 is essential for its function in autophagy and development and is responsible for interaction with MoAtg6. The MoAtg14-ΔCCD mutant lacking the coiled-coil domain had a phenotype similar to that of the Δ*Moatg14* mutant. However, the MoAtg14-ΔC and the MoAtg14-ΔN mutants, which expressed truncated MoAtg14 lacking the C-terminal or N-terminal half, showed no obvious defect in colony morphology, conidiogenesis, pathogenicity or autophagic activity.

The sequence identity between MoAtg14 and yeast Atg14 is extremely low. In *S. cerevisiae*, Δ*atg14* shows defects in viability and autophagy. To investigate the functional homology between Atg14 and MoAtg14, the complementation vector pYA14 containing the full-length yeast *ATG14* was transformed into the Δ*Moatg14* mutant to generate the Δ*Moatg14*::*ATG14* strain. Unfortunately, the Δ*Moatg14*::*ATG14* strains showed sparse aerial hyphae on CM medium similar to the mutant Δ*Moatg14*. In addition, no autophagic bodies accumulated in the vacuoles of the Δ*Moatg14*::*ATG14* strains when it was grown in sterile distilled water (starvation conditions) containing 2 mM PMSF for 4 h (data not show). Yeast Atg14 could not complement the autophagic defects of the Δ*Moatg14* mutant. We deduced that Moatg14 may have different biological functions in *M. oryzae*.

Conidiation is essential for the rice blast fungus to carry out its infection cycle. Conidiation was impaired in many autophagy-deficient mutants, including *M. oryzae* Δ*Moatg1, 4, 5, 8, 9*^9^,^18^,^19^,^21^,^22^, *F. graminearum* Δ*Fgatg8* and Δ*Fgatg15*^23^,^25^, *C. lindemuthianum* Δ*atg1*/Δ*clk1*^28^, *C. orbiculare* Δ*atg26*^26^, *Ustilago maydis* Δ*atg1* and Δ*atg8*^30^. In our present study, Δ*Moatg14* showed sparse aerial hyphae and reduced conidiation. These data indicated that autophagy plays key roles in aerial hyphal growth and conidiophore differentiation. It has been reported that sucrose or glucose could restore conidiation of the Δ*Moatg8* mutant to a level comparable to the wild type by external supplementation of carbohydrates^22^. Although external supplementation of carbohydrates, including G6P and sucrose, alleviated the conidiation defects of the Δ*Moatg14* mutant, the recovery of the conidiation defects is not comparable to that of the wild type. In yeast, Atg14 is required for membrane trafficking pathways^33^. In addition, yeast Atg14 could not complement the defects of the Δ*Moatg14* mutant. We proposed that MoAtg14 might play other different biological roles beyond autophagy in *M. oryzae*, such as membrane trafficking pathway and so on. Therefore, it is necessary to explore the additional functions beyond autophagy of MoAtg14 in *M. oryzae*.

In addition to reduced conidia in Δ*Moatg14*, there was a loss of pathogenicity. At present, the evidence suggests that delayed degradation of the glycogen, less lipid bodies and reduced turgor pressure result in defects in pathogenicity upon loss of autophagy in *M. oryzae*^10^. Our data provide evidence that the reasons for the loss of pathogenicity in Δ*Moatg14* are consistent with those for the other autophagy deficient mutants (Δ*Moatg1, 4, 5, 8, 9*) in *M. oryzae*.

Atg8 can localize to autophagosomes and be internalized in vacuoles after the fusion of autophagosomes and vacuoles. Autophagy can be visualized directly by fluorescent marker tagged Atg8^41^. A bright punctate structure representing the PAS is observed when the mycelia of the wild-type strain Guy11 expressing GFP-MoAtg8 are grown in nutritious conditions. However, the vacuole lumen is stained with fluorescence, which reflects the induction of autophagy when the same mycelia are starved for 4 h. Under the same starvation conditions, no movement of GFP-MoAtg8 to the vacuolar lumens were observed when autophagy was induced in the Δ*Moatg14* mutant. Loss of MoAtg14 prevents MoAtg8 from leaving the PAS structure in *M. oryzae*. In addition, the GFP-MoAtg8 proteolysis assays in the Δ*Moatg14* mutant confirmed the fluorescent visualization of GFP-MoAtg8. No autophagic bodies were observed in the vacuole lumens of the Δ*Moatg14* mutant using microscopy assays. These results are in accord with the movement and proteolysis of GFP-MoAtg8 in the Δ*Moatg1* mutant, in which the autophagy was fully blocked^18^,^42^. It has been reported that most of autophagy-related proteins accumulate at the PAS and generate autophagosomes. Autophagy-related proteins are classified into several different groups at the different steps of the autophagy pathway. MoAtg1 has an effect on the initiation of autophagy and is involved in the building of the PAS. MoAtg14, an autophagy-specific regulator of the PI3-K complex, contributes to autophagosome formation of autophagy and mediates the transfer of other autophagy-related proteins to PAS. In addition to the changes in the GFP-MoAtg8 localization and proteolysis, the Δ*Moatg14* mutant showed defects in autophagic cell death. The FDA staining assays showed that many of the Δ*Moatg14* conidia contained FDA signals during the appressorial development. Death of the conidia was prevented upon loss of autophagy in the Δ*Moatg14* mutant. It has been reported that autophagic cell death is important for fungal developmental biology and pathogenesis^8^,^43^. Our data provide evidence that MoAtg14 is an important factor of autophagy in *M. oryzae*.

MoAtg14 has only one coiled-coil domain. MoAtg14 interacts with MoAtg6 through this coiled-coil domain, and this interaction is required for autophagy. The coiled-coil domain of MoAtg14 is related to autophagy and pathogenicity. However, the N-terminus or C-terminus of MoAtg14 is not essential for autophagy and pathogenicity. The Barkor/Atg14(L) autophagosome-targeting sequence (BATS) domain exists in the C-terminal half of mammalian Atg14. The BATS domain preferentially binds to the highly curved membranes containing PI3P and is proposed to target the PI3-K complex efficiently to the isolation membrane^37^,^44^. In yeast, there are three predicted coiled-coil domains within Atg14p. Recent studies show that the C-terminus controls the size of the autophagosome although there is no conserved domain in yeast Atg14^35^,^39^. Therefore, it is interesting to search for the potential functions of every part of MoAtg14 in *M. oryzae*.

The class III PI3-K complex is the critical regulator of autophagy. In yeast, there are two distinct PI3-K complexes as follows: the type I complex consisting of Vps34, Vps15, Atg6/Vps30 and Atg14, and the type II complex containing Vps34, Vps15, Atg6/Vps30, and Vps38. Atg14 and Vps38 are specifically integrated into type I and type II complexes, respectively. In addition, Atg14 and Vps38 play key roles in determining the function of PI3-K complexes^33^,^45^. The two distinct PI3-K complexes have been studied in mammals and found to be similar to those in yeast. The counterparts of Vps34, Vps15, and Vps30/Atg6 are Vps34, p150, and Beclin 1, respectively. The mammalian UV irradiation resistance-associated gene (UVRAG) has been identified as identical to yeast Vps38. Researchers found another PI3-K complex consisting of Vps34, Vps15, Beclin 1, UVARG and Rubicon^46^,^47^,^48^. In *M. oryzae*, MoAtg14 has been confirmed to play key roles in autophagy, and it interacts with MoAtg6 via the conserved coiled-coil domain. Hence, we deduced that the type I PI3-K complex might be conserved in *M. oryzae*. Although the conserved functions of MoAtg14 have been confirmed in our studies, it is necessary to solve a problem on the mechanism of MoAtg14 bridges MoAtg6 to form the type I PI3-K complex in *M. oryzae*. The other protein, MGG_13375 (designated as MoVps38), shows higher similarity to mammalian UVRAG protein (a counterpart of the mammalian Vps38) and has the conserved coiled-coil domain. It implied that MGG_13375 might represent the fungal ortholog of Vps38. In addition, MoVps38 interacted with MoAtg6 using the yeast two hybrid assays (data not show). We speculated that the other distinct PI3-K complex members might exist in *M. oryzae*. Unfortunately, the null mutant of MoVps38 was not obtained in the wild-type strain Guy11 by screening hundreds of transformants. MoVps38 might play key roles and be a viable factor in *M. oryzae*. Similar results were shown in *Sordaria macrospora*. SmVps15 and SmVps34, the homologs of yeast Vps15 and Vps34 in the class III PI3-K complex, were identified in *S. macrospora*^49^. However, the authors could not generate the homokaryotic knockout mutant of SmVps15 and SmVps34. This result suggested that Smvps34 and Smvps15 are required for viability. It is necessary to ascertain the components of the type II PI3-K complex or other types of the PI3-K complex.

In conclusion, this study describes the functions of MoAtg14 in *M. oryzae*, a hitherto uncharacterized protein. Based on the sequence alignment and functional similarity, MoAtg14 is the homolog of yeast Atg14. During the development of *M. oryzae*, MoAtg14 plays key roles in aerial hyphae, conidiation, degradation of glycogen, numbers of lipid bodies, autophagic cell death and pathogenicity. The autophagy process is blocked in the Δ*Moatg14* mutant. Loss of MoAtg14 prevents MoAtg8 from leaving the PAS structures in *M. oryzae*. MoAtg14 could co-localize with MoAtg8. MoAtg14 directly interacted with MoAtg6 through the coiled-coil domain. Further work is needed to determine the diverse functions beyond autophagy of MoAtg14 in *M. oryzae*.

## Experimental Procedures

### Fungal strains and culture conditions

The wild-type strain Guy11 and the mutants of *M. oryzae* were cultured on complete medium (CM) at 25 °C with a 12-h photophase, as described previously^50^. Other media included V8 medium, OMA medium and MM as reported previously^42^. For DNA and RNA extractions, mycelia were harvested from 7-day-old cultures grown in liquid CM at 25 °C with shaking at 150-rpm. General molecular biology techniques for nucleic acid analysis were performed according to standard protocols^51^. Phenotypic analyses including screens for the formation of conidia, conidiophore formation, conidia germination, appressoria formation, and pathogenicity assays were carried out as reported previously^42^.

### Generation of the knockout vector and mutants

The *MoATG14* gene deletion vector was constructed using a strategy based on double-joint PCR^52^. The 1.0 kb upstream and 1.2 kb downstream flanking sequences of the *MoATG14* were amplified from Guy11 genomic DNA using the primers ATG14up-1/2 and ATG14dn-1/2, respectively. A 1.4 kb *HPH* cassette was cloned from pCB1003 using the primers HPH-1/2. The three amplicons were joined together in the second round of PCR, the product of which served as the template for the final construct amplification using the nested primers ATG14-N-1/2. The double-joint PCR product was inserted into the *Sal*I/*Xba*I sites of pCAMBIA1300 to generate the targeted-gene-deletion vector (Fig. 2A). The vector was introduced into the wild-type strain Guy11 via *Agrobacterium tumefaciens*-mediated transformation (ATMT) as described previously^53^. After PCR screening using ATG14-C-1/2, the putative MoAtg14 null mutants were confirmed by Southern blot analysis. For the complementation assay, a 4.0-kb PCR product containing a 2.0-kb upstream sequence, a full-length *MoATG14* gene coding region, and a 0.6-kb downstream sequence was amplified from Guy11 genomic DNA using the primers ATG14C-1/2, and inserted into a modified pCAMBIA1300 vector, which contained a geneticin resistance gene. The resulting vector for the complementation assay was randomly inserted into the genome of the Δ*Moatg14* mutant using the ATMT method. Southern blot analysis was carried out to verify the single-copy integration according to the manufacturer’s instructions for the Digoxigenin (DIG) High Prime DNA Labeling and Detection Starter Kit I (Roche, USA). Quantitative RT-PCR was performed as described previously^15^.

### Construction of the pGFP-MoAtg14, pMoAtg14-ΔC, pMoAtg14-ΔN and pMoAtg14-ΔCCD vectors

The cDNA fragment containing the full coding sequence of the *MoATG14* gene was cloned from the 24 h-appressoria cDNA library^54^, and inserted to the *Xba*I site of the vector pKD5-GFP using the primers Atg14FL-F/R to generate the vector pGFP-MoAtg14. The C-terminal, N-terminal, and coiled-coil domain (CCD) fragments were amplified from the *MoATG14* cDNA using the primers Atg14C-F/R, Atg14N-F/R, and Atg14CCD-F/R, respectively. The amplified fragments were inserted into the *Sma*I/*Xba*I, *Xba*I, and SmaI/*Bam*HI sites of the vector pKD61^42^ to generate the vectors pMoAtg14-ΔC, pMoAtg14-ΔN, and pMoAtg14-ΔCCD, respectively. The vectors were introduced into the mutant Δ*Moatg14* via ATMT.

### Protein manipulation and immunoblot analysis

Total protein extractions and SDS-PAGE were carried out as reported previously. For the GFP-MoAtg8 proteolysis assay, the pGFP-MoAtg8 vector was transformed into Guy11 and the mutants using the ATMT method as previously described^42^. Transformants expressing GFP-MoAtg8 were selected to detect full-length GFP-MoAtg8 and free GFP with anti-GFP western blots. The total protein content for the western blots was quantified using a Bradford Protein Assay Kit (Biyuntian, China).

### Staining and Microscopy

Conidial suspensions (1 × 10^5^/ml) were incubated on hydrophobic films to form appressoria in a humid chamber at 25 °C. Glycogen and lipid bodies were observed during development at 0 h, 4 h, 8 h and 24 h post-incubation with KI/I_2_ and Nile red staining as previously described^18^,^40^. To detect autophagic cell death, fluorescein diacetate (FDA), a viability stain for cells, was used. The samples were treated with 25 μg/ml FDA staining solution and incubated at room temperature for 4 to 5 minutes in the dark. FDA signals were analyzed with fluorescent microscopy.

Fluorescent microscopy observations were carried out as previously described^42^. Light and epifluorescence microscopic examinations were performed using an Eclipse 80i microscope (Nikon) and a ZEISS LSM780 inverted confocal microscope (Carl Zeiss Inc.).

### Yeast two-hybrid interaction

The CDS of MoAtg6 was cloned into pGBKT7 (Clontech, USA) to form the bait constructs. Similarly, MoAtg14 was cloned into pGADT7. The construct strategy used the In-Fusion HD Cloning Kit (Clontech, USA) and the primer pairs were listed in Table S1. The resulting prey and bait constructs were confirmed by sequencing analysis and co-transformed in pairs into the yeast strain AH109 according to the instructions of the Matchmaker Gal4 Two-Hybrid System 3 (Clontech, USA). The positive transformants on SD-Leu-Trp medium were then tested on SD-Ade-His-Leu-Trp medium. The positive and negative control strains used in the assay were from the cloning kit.

## Additional Information

**How to cite this article**: Liu, X.-H. *et al*. Autophagy-related protein MoAtg14 is involved in differentiation, development and pathogenicity in the rice blast fungus *Magnaporthe oryzae. Sci. Rep.* **7**, 40018; doi: 10.1038/srep40018 (2017).

**Publisher's note:** Springer Nature remains neutral with regard to jurisdictional claims in published maps and institutional affiliations.

## Supplementary Material

Supplementary Information
