# Supplementary material for: Autophagy-related protein MoAtg14 is involved in differentiation, development and pathogenicity in the rice blast fungus Magnaporthe oryzae
Source: Sci Rep. 2017 Jan 9;7:40018. doi: 10.1038/srep40018 (PMC5220305; doi:10.1038/srep40018)
Supplement: Supplementary Information [file srep40018-s1.pdf]

Autophagy-related protein MoAtg14 is involved in differentiation, development and pathogenicity in the rice blast fungus *Magnaporthe oryzae*

Xiao-Hong Liu[1], Ya-Hui Zhao[1], Xue-Ming Zhu[1], Xiao-Qing Zeng[4], Lu-Yao Huang[1], Bo Dong[5], Zhen-Zhu Su[1,3], Yao Wang[1], Jian-Ping Lu[2], Fu-Cheng Lin[1]*

Table S1 Primers in this study

Name	Sequence (5'-3')
ATG14up-1	CAGTGTTGATGAGGTTGGGA
ATG14up-2	CATTCATTGTTGACCTCCACTAAAGGGCAGAGTAGTCAGGAAA
ATG14dn-1	GGGCAAAGGAATAGAGTAGATGATAGAATTGTCCCCTGAGCCC
ATG14dn-2	ACATCGTTTGTTTCCCGTGA
ATG14-N1	ACGTCGACTCCTGTGAATCTTCTGGGTG
ATG14-N2	CGTCTAGAGGGTGATAGCGTCCTGGTTG
ATG14-C1	CTTGGTCGTCGTCAATCTCG
ATG14-C2	CTGTCCGCAGCCCTTGTTAG
ATG14pb-1	ACGTCGACTCCTGTGAATCTTCTGGGTG
ATG14pb-2	AGGGCAGAGTAGTCAGGAAA
Atg14FL-F	GAGCTGTACAAGTCTAGAATGTCATGCTATATCTGCGG
Atg14FL-R	CTGCAGGTCGACTCTAGAAGTCCAAGCATCATCCTCCA
ATG14N-F	GCTCTAGAATGTCATGCTATATCTGCGG
ATG14N-R	AATCTAGATGCAGCCTTGCGAGCTGCGG
ATG14CCD-F	AACCCGGGATGCGCTCATCCCCCGCAGCTCG
ATG14CCD-R	AGGTCGACATCCCAGTCGGCCCTGGTTG
ATG14C-F	AACCCGGGAATATTGCGGCAACCAGGGC
ATG14C-R	AGTCTAGAAGTCCAAGCATCATCCTCCA
ATG6-BDF	CATGGAGGCCGAATTCATGATGTTTTGCCAAAAATGCCGGAC
ATG6-BDR	GCAGGTCGACGGATCCTTAGGTCGAGCTTGAGCCCAAAACCT
ATG6CCD-BDF	CATGGAGGCCGAATTCATGGTCGAAGGCCTGCGCAAGCGCCT
ATG6CCD-BDR	GCAGGTCGACGGATCCATACACGTTTGTCCGCTGCAGAGACT
ATG14-ADF	GGAGGCCAGTGAATTCATGTCATGCTATATCTGCGGACGCGG
ATG14-ADR	CGAGCTCGATGGATCCTCAAGTCCAAGCATCATCCTCCAGCA
ATG14CCD-ADF	GGAGGCCAGTGAATTCATGCGCTCATCCCCCGCAGCTCG
ATG14CCD-ADR	CGAGCTCGATGGATCCATCCCAGTCGGCCCTGGTTG
ATG14N-ADF	GGAGGCCAGTGAATTCATGTCATGCTATATCTGCGGACGCGG
ATG14N-ADR	CGAGCTCGATGGATCCTGCAGCCTTGCGAGCTGCGG
ATG14C-ADF	GGAGGCCAGTGAATTCATGAATATTGCGGCAACCAGGGC
ATG14C-ADR	CGAGCTCGATGGATCCTCAAGTCCAAGCATCATCCTCCAGCA
FgAtg14C-F	CCCGGGAAGTCTAGAATGGACTGTGATATCTGTCATC
FgAtg14C-R	CAGGTCGACTCTAGATCATCTATTCTTGACCCTCATC
TrAtg14C-F	CCCGGGAAGTCTAGAATGGAATGCGACATCTGCCACC
TrAtg14C-R	CAGGTCGACTCTAGATCATCTGCTCTTGACCTTCATC

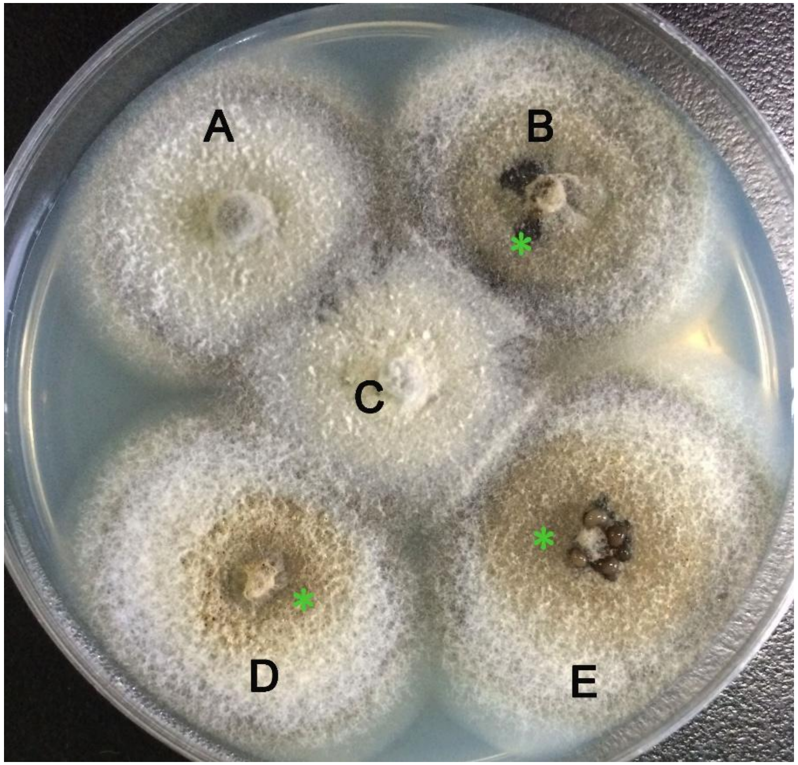

FgAtg14 to the Δ*MoAtg14* mutant

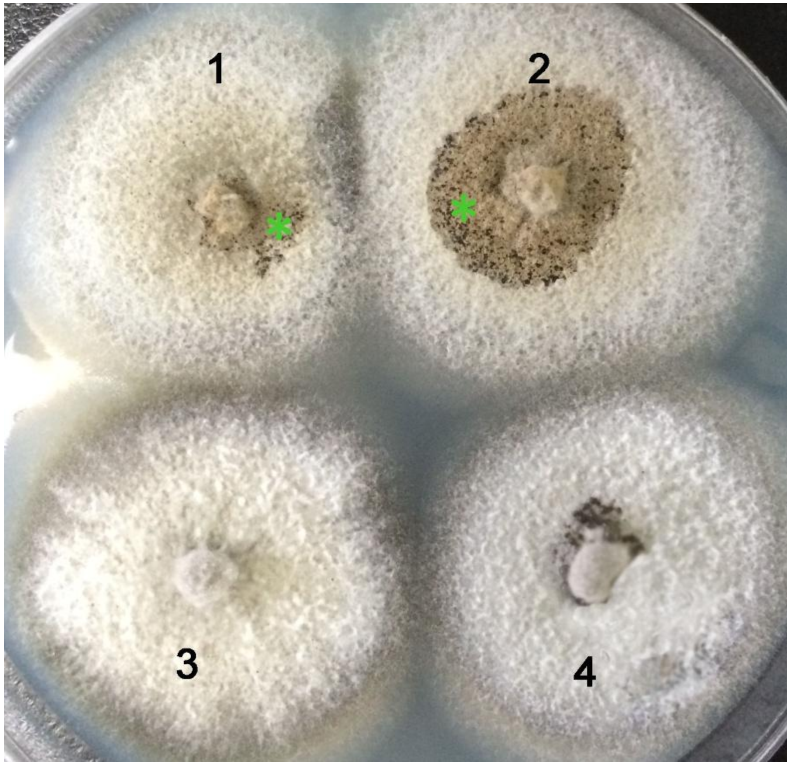

TrAtg14 to the Δ*MoAtg14* mutant

Figure S1 Complementation assays of FgAtg14 and TrAtg14

Left, introduction FgAtg14 to the Δ*MoAtg14* mutant, A & C are recovered, and similar to the wild type.
Right, introduction TrAtg14 to the Δ*MoAtg14* mutant, 3 & 4 are recovered, and similar to the wild type.
* the typical characteristics of the Δ*MoAtg14* mutant.

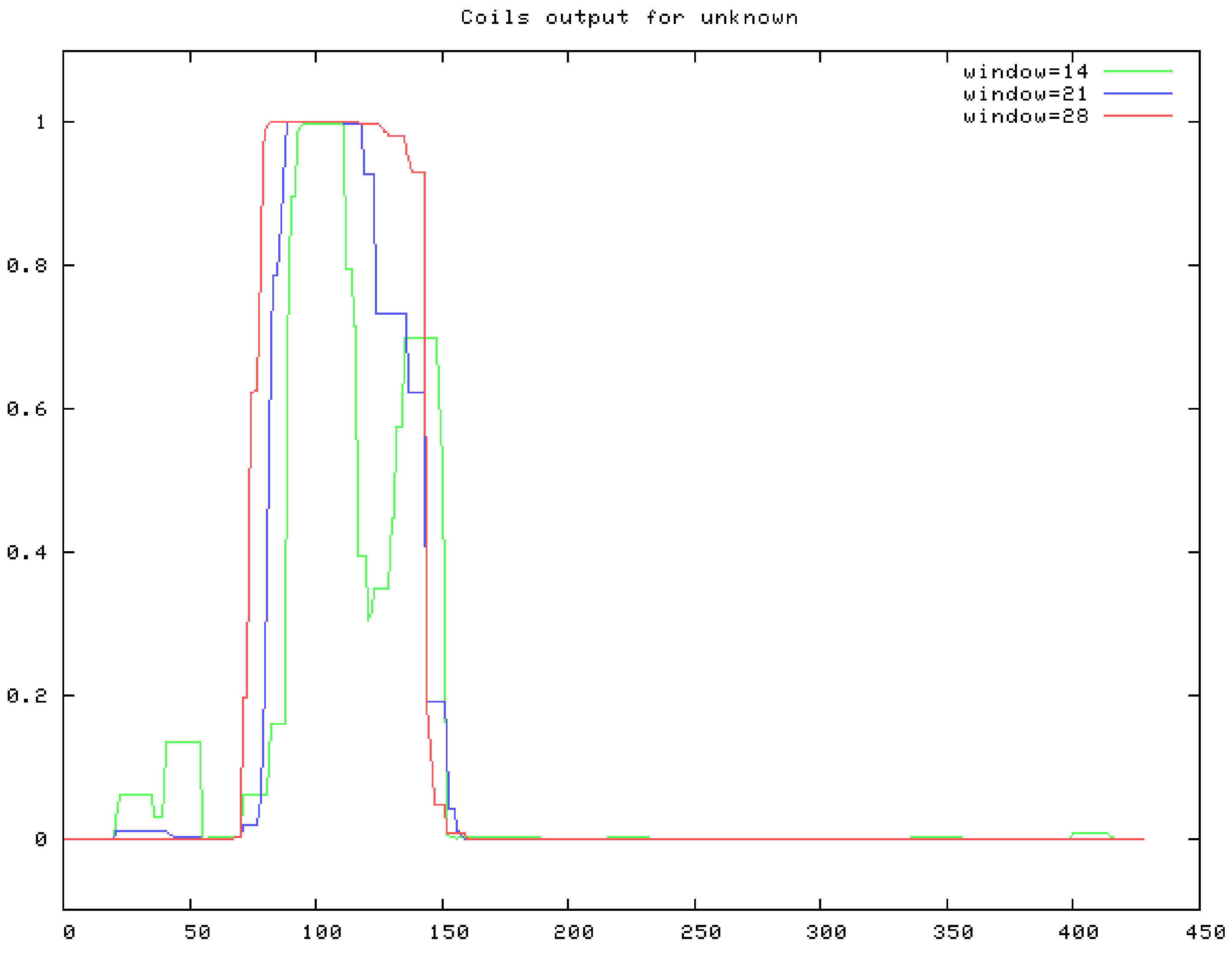

Figure S2 The coiled-coil domain was predicted within the MoAtg14.

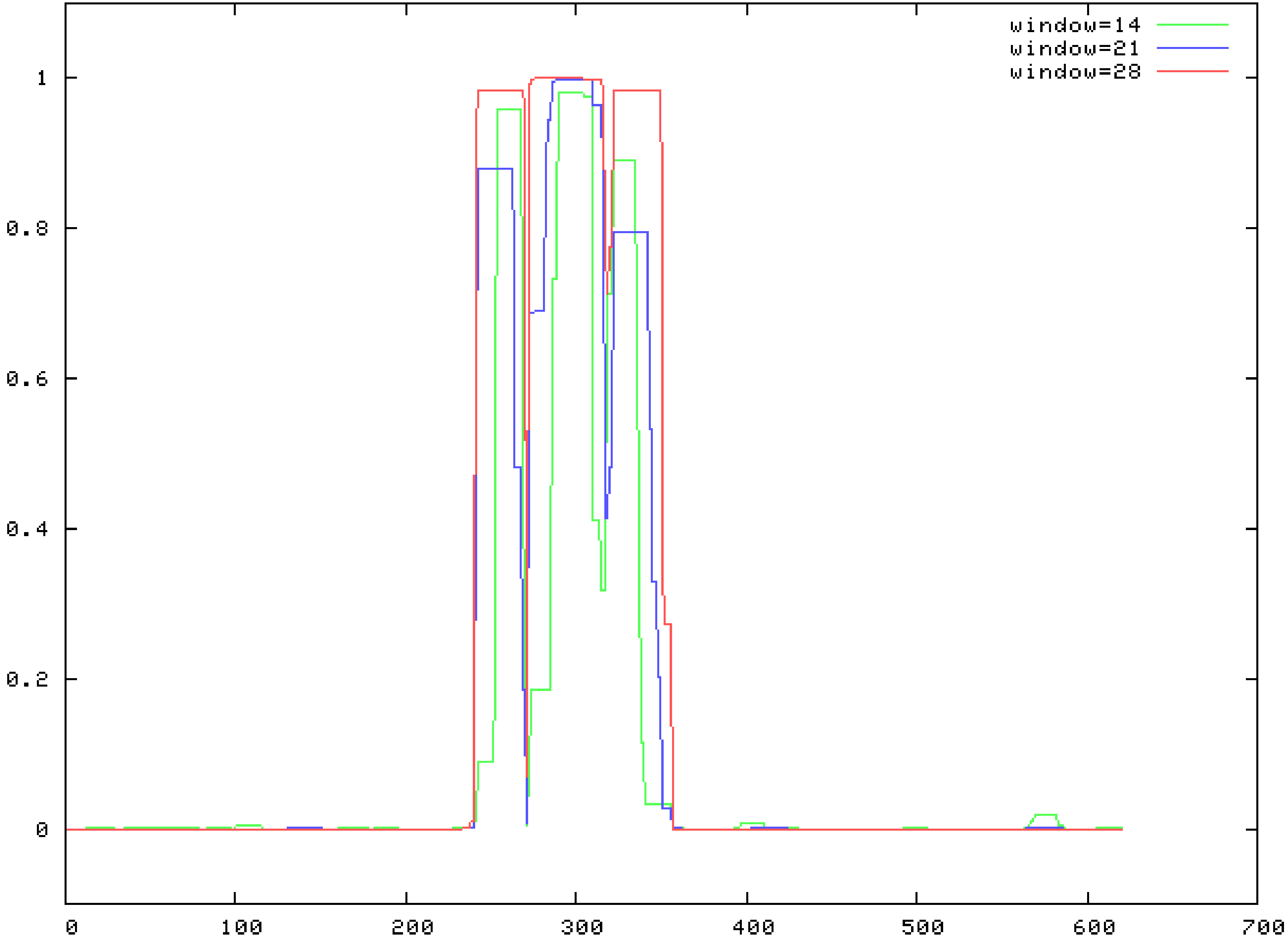

Figure S3 The coiled-coil domain was predicted within the MoVps38.

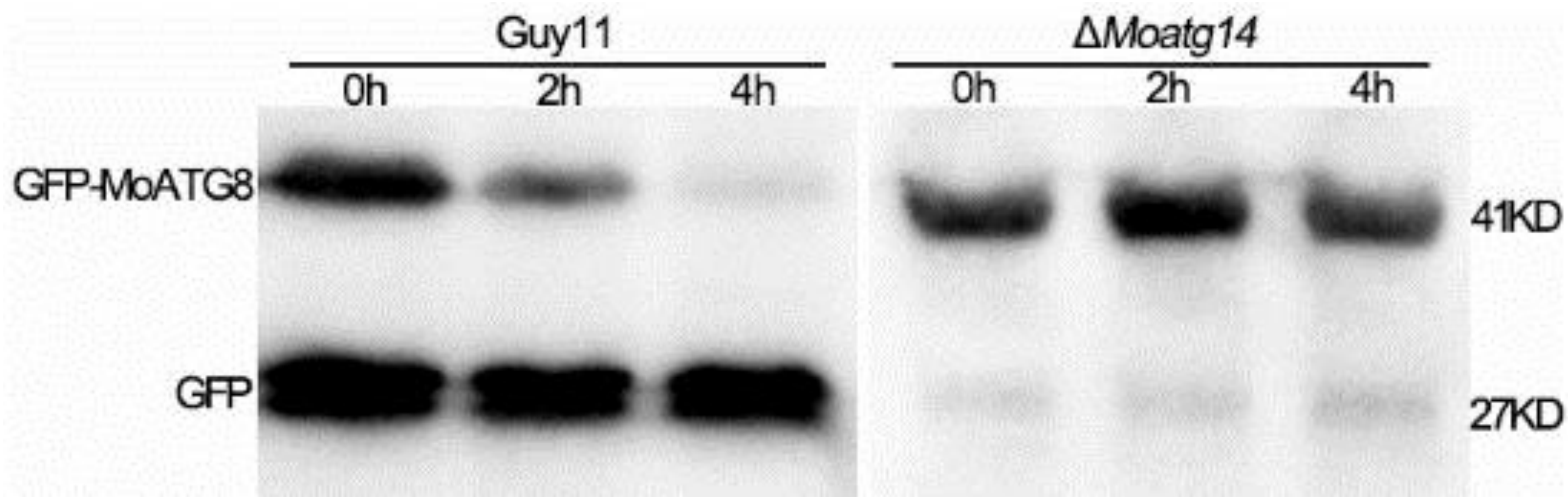

Figure S4 GFP-MoAtg8 proteolysis assays of Guy11 and Δ*MoAtg14*. Mycelia were cultured at 25 °C for 48 h in CM liquid medium continuously shaken at 150 rpm. Autophagy was induced after 2 and 4 h of nitrogen starvation. The mycelia were collected at the indicated times, and the mycelial extracts were analyzed by anti-GFP western blotting.

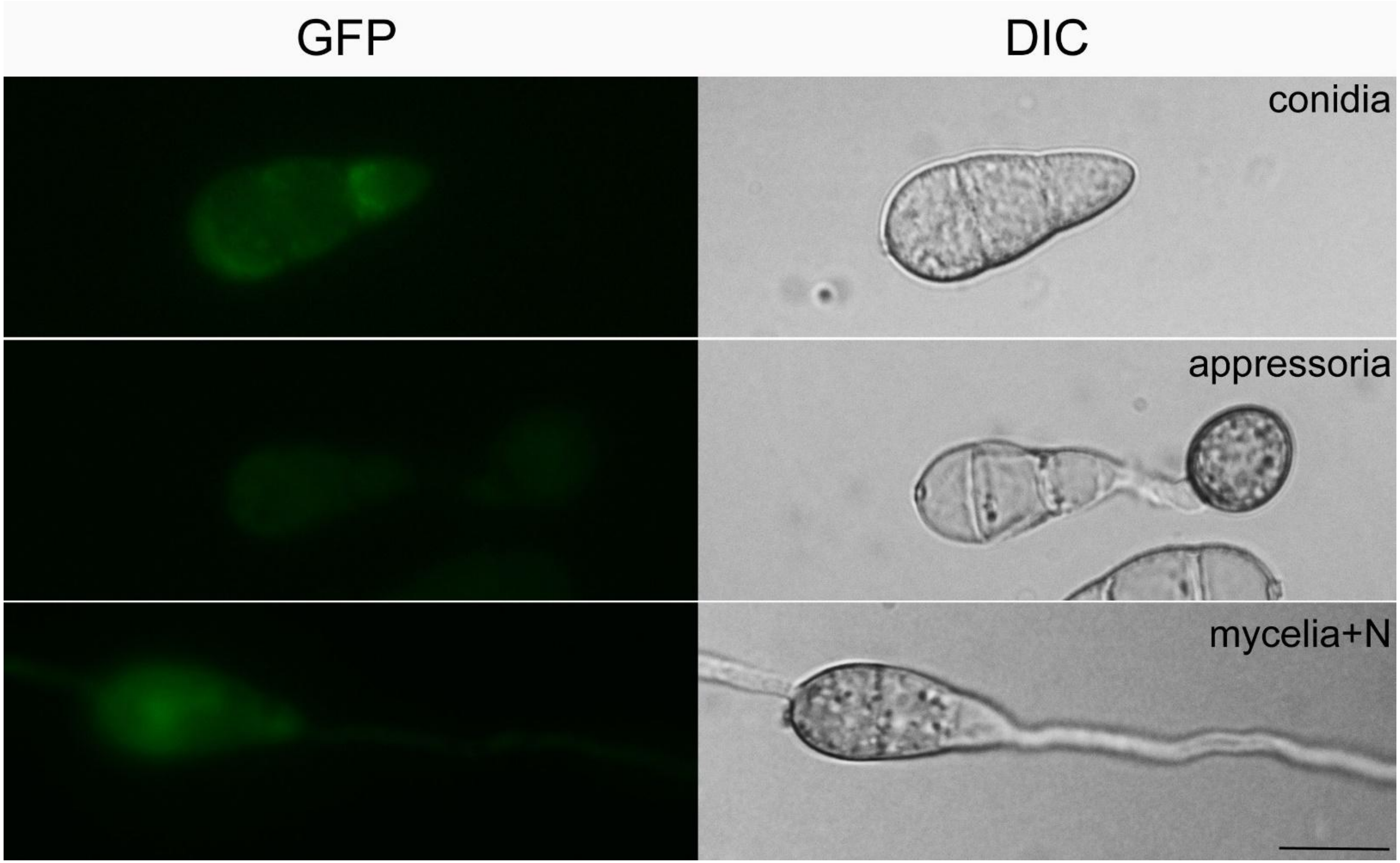

Figure S5 Subcellular localization of GFP-MoAtg14 in conidia, appressoria and mycelia. Scale bar = 10 μm.
